# IBPGNET: lung adenocarcinoma recurrence prediction based on neural network interpretability

**DOI:** 10.1093/bib/bbae080

**Published:** 2024-03-31

**Authors:** Zhanyu Xu, Haibo Liao, Liuliu Huang, Qingfeng Chen, Wei Lan, Shikang Li

**Affiliations:** Department of Thoracic and Cardiovascular Surgery, The First Affiliated Hospital of Guangxi Medical University, Nanning, Guangxi Zhuang Autonomous Region 530021, China; School of computer, Electronic and Information, Guangxi University, Nanning, Guangxi Zhuang Autonomous Region 530021, China; Department of Thoracic and Cardiovascular Surgery, The First Affiliated Hospital of Guangxi Medical University, Nanning, Guangxi Zhuang Autonomous Region 530021, China; School of computer, Electronic and Information, Guangxi University, Nanning, Guangxi Zhuang Autonomous Region 530021, China; School of computer, Electronic and Information, Guangxi University, Nanning, Guangxi Zhuang Autonomous Region 530021, China; Department of Thoracic and Cardiovascular Surgery, The First Affiliated Hospital of Guangxi Medical University, Nanning, Guangxi Zhuang Autonomous Region 530021, China

**Keywords:** lung adenocarcinoma, neural network interpretability, PSMC1 and PSMD11, multi-omics data, recurrence prediction

## Abstract

Lung adenocarcinoma (LUAD) is the most common histologic subtype of lung cancer. Early-stage patients have a 30–50% probability of metastatic recurrence after surgical treatment. Here, we propose a new computational framework, Interpretable Biological Pathway Graph Neural Networks (IBPGNET), based on pathway hierarchy relationships to predict LUAD recurrence and explore the internal regulatory mechanisms of LUAD. IBPGNET can integrate different omics data efficiently and provide global interpretability. In addition, our experimental results show that IBPGNET outperforms other classification methods in 5-fold cross-validation. IBPGNET identified PSMC1 and PSMD11 as genes associated with LUAD recurrence, and their expression levels were significantly higher in LUAD cells than in normal cells. The knockdown of PSMC1 and PSMD11 in LUAD cells increased their sensitivity to afatinib and decreased cell migration, invasion and proliferation. In addition, the cells showed significantly lower EGFR expression, indicating that PSMC1 and PSMD11 may mediate therapeutic sensitivity through EGFR expression.

## INTRODUCTION

According to the Global Cancer Observatory, lung cancer has the second highest incidence of all cancers worldwide. With a mortality rate of 18%, it is the leading cause of cancer death in both men and women [1]. Lung adenocarcinoma (LUAD) is the most common type of pathology, accounting for around 40% of all lung cancer cases worldwide [2]. According to the American Cancer Society (2022), approximately 56% of all new lung cancer cases are diagnosed at a regional or distant stage after cancer has spread to nearby lymph nodes or other body parts [3]. It is important to note that early detection of LUAD can significantly improve treatment success and survival. Accurate diagnosis, personalized treatment and recurrent disease monitoring have plagued the medical community due to the heterogeneity of LUAD disease [4]. Therefore, identifying molecular markers and biological pathways involved in the recurrence and metastasis of LUAD is crucial for disease understanding and targeted therapy development, consequently improving diagnosis, prognosis and survival.

Multi-omics data, such as genomics, proteomics and metabolomics, have accumulated with the development of high-throughput sequencing technologies [5, 6]. As a representation of information at the individual and cellular levels, these omics data are expected to reveal further gene regulation mechanisms, cellular metabolic processes and molecular response pathways, providing more insights into cancer pathogenesis [7–9]. The available multi-omics data and rich clinical information can help understand LUAD recurrence mechanisms and identify its associated driver genes, pathways and compounds.

Deep learning has achieved excellent results in LUAD prognostic studies due to its excellent nonlinear fitting ability [10, 11]. However, due to the ‘black box’ property of deep learning models, there is no insight into their internal decision processes, which is fatal for subsequent model evaluation and biological interpretation [12, 13]. Therefore, balancing the accuracy and interpretability of the deep learning model is very important in biomedical research. Many interpretable methods, such as LIME [14], RISE [15], Grad-CAM [16] and DeepLIFT [17], have been spawned to improve the interpretability of deep learning models. Besides, some methods inspired by biological systems have been successfully applied to cancer classification prediction by constructing neural networks with biological information [18–21].

In this study, we propose a new computational framework, Interpretable Biological Pathway Graph Neural Networks (IBPGNET), based on the pathway hierarchy relationship to predict LUAD recurrence and explore the internal regulatory mechanisms of LUAD. In IBPGNET, the pathway hierarchy is compressed into a neural network for capturing gene-to-phenotype regulatory relationships. Then, a graph neural network is designed to solve network sparsity by predicting latent pathway relationships. We investigated the expression levels of PSMC1 and PSMD11, two key genes in the IBPGNET network, in five different LUAD cells. Following this, we created stable knockdown models of PSMC1 and PSMD11 using two LUAD cell lines and evaluated their cell migration, invasion and proliferation using functional assays. We also assessed the expression of EGFR and the cells’ sensitivity to varying concentrations of afatinib. This study offers a novel approach to exploring the molecular mechanisms underlying LUAD and identifies potential therapeutic targets with clinical applications in disease diagnosis and treatment.

## MATERIALS AND METHODS

### Overview of IBPGNET

IBPGNET is an interpretable network for recurrence prediction and related biomarker discovery in LUAD. The structure is shown in Figure S1. The IBPGNET network structure is constructed based on the Reactome pathway hierarchy relationships [22], where nodes represent specific biological entities, edges represent entity relationships and the connections represent the network flow from low-level biological entities to high-level biological entities. The workflow of IBPGNET can be summarized into four components.

(i) Data preprocessing, where noise removal and feature selection were performed to remove the artificially introduced noise caused by sequencing equipment.

(ii) The construction of an interpretable network with biological information for the model’s self-interpretation by Reactome biological pathway hierarchies.

(iii) Bio-entity relationship prediction using graph neural network; since biological entity relationships have not been thoroughly mined, the graph neural network is used to learn representations and predict entities’ linkage with updated information for biological link discovery.

(iv) Interpretability of the model, importance scores of multi-omics data and biological entities in the network by backpropagation are utilized to identify biomarkers associated with LUAD recurrence.

Data preprocessing: due to the limitations of sequencing technology, some outliers and missing data exist in multi-omics data, affecting the model’s prediction performance. In this experiment, the copy number variant (CNV) and somatic mutation data of LUAD were obtained from the Xena TCGA Pan-Cancer web [23]. The CNV data consisted of GISTIC-focal score by gene type, and the simple nucleotide variation (SNV) data constituted VarScan2 Variant Aggregation and Masking type. Clinical data for LUAD was downloaded from the TCGA database [24], and only samples with all three data were retained. The copy number variation was divided into two subsets: copy number amplification (AMP_CNV) and copy number deletion (DEL_CNV). In addition, low variance features and a large number of missing values were considered noisy and filtered out.

Although the integrative analysis of multi-omics data can provide more insights into cancer treatment and diagnosis, the prediction performance is degraded by overfitting during training in neural networks due to the high-dimensional and small-sample characteristics of multi-omics data. Besides, many non-relevant features in the omics data can affect the prediction results. Therefore, in the data preprocessing stage, the chi-square test [25] was used to select features from the different omics data separately. The top 3000 significant variables by *P*-value were selected as pre-training features. The details of the data are shown in Table 1.

**Table 1:** Summary of datasets

Dataset	Categories	Number of features of SNV, AMP_CNV and DEL_CNV	Number of training features of SNV, AMP_CNV and DEL_CNV
LUAD	Recurrence: 134	18 498, 19 645, 19 645	3000, 3000, 3000
	Non-recurrence: 371		

### Construction of interpretable networks with biological information

The backbone network of IBPGNET is a multilayer pathway network constructed according to the Reactome pathway hierarchy relationship. The nodes represent specific biological entities, and the edges represent entity-to-entity relationships, such as binding, activation, translocation, degradation and catalysis. IBPGNET uses a five-layer pathway network, and the connections between entities flow from lower-level biological entities to higher-level biological entities. Some entities without successor nodes were removed. Let *H* = $\left[h1,h2,\dots, h5\right]$ denote the layer’s number in the hidden layers and ${h}_i^j$ represent the *j*th node on the *i*th layer. ${H}_{\mathrm{connet}}\left({h}_{i-1}^j,{h}_i^k\right)$ is the connection from the *j*th node on the (*i*−1)th layer to the *k*th node on the *i*th layer, which is defined as follows:


(1) \begin{equation*} {H}_{\mathrm{connet}}\left({h}_{i-1}^j,{h}_i^k\right)=\left\{\ \begin{array}{c}1,\ \mathrm{if}\kern0.5em {h}_i^k\kern1.25em \mathrm{next}\left({h}_{i-1}^j\right)\\{}\kern0.75em 0,\kern1.00em \mathrm{otherwise}\end{array}\ \right. \end{equation*}

where $\mathrm{next}\left({h}_i^j\right)$ denotes the set of successor nodes of the *j*th neuron on the *i*th layer. If ${H}_{\mathrm{connet}}\left({h}_{i-1}^j,{h}_i^k\right)$ equals 1, there is a connection between node *j* and *k*; otherwise, there is no connection. If ${h}_{i-1}^j$ has no successor node, it will be removed. In addition, when the five-layer network is constructed, the nodes that do not conform to the five-layer structure will also be removed.

In the forward propagation of the network, let $M=\big[{M}_1,{M}_2,\dots, {M}_5\big]$ represent the hierarchical relationship of paths at each layer. Multilayer perceptron (MLP) is used to predict cancer recurrence. The rule in forward propagation is as follows:


(2) \begin{equation*} f\left({X}_{i+1}\right)=\mathrm{\sigma} \left({\left({M}_i\ast{W}_i\right)}^T\ast{X}_i+{b}_i\right)\ i=\left[1,2,\dots, 5\right] \end{equation*}

(3) \begin{equation*} \hat{y_{\mathrm{i}}}=\mathrm{MLP}\left(f\left({X}_6\right)\right)\qquad\qquad\qquad\qquad\quad\, \end{equation*}

where σ denotes the tanh activation function. ${M}_i$ denotes the path matrix of the *i*th layer. ${W}_i$ denotes the trainable parameter of the *i*th layer. ${X}_i$ denotes the input of the *i*th layer.

Since there is a positive and negative sample imbalance in multi-omics data, the weight of the class is added to the loss function to balance the classes. The larger the class weight, the more penalties and updates are applied to the model coefficients. The loss function of the network is defined as follows:


(4) \begin{equation*} \mathrm{Loss}=\frac{1}{N}\ \sum_{i=1}^N\left[-\right({w}_1\left({y}_i\ast \log \left(\hat{y_i}\right)\right)+{w}_0\left(\left(1-{y}_i\Big)\ast \log \left(1-\hat{y_{\mathrm{i}}}\right)\right)\right)\Big] \end{equation*}

where $w1$ represents the class weight of positive samples. $\mathrm{w}0$ represents the class weight of negative samples. ${y}_i$ and $\hat{y_i}$ represent the target class’s actual value and predicted probability, respectively.

### Pathway hierarchy relationship prediction based on graph convolutional neural network

The Reactome pathways database represents a knowledge database of molecular interactions. However, the current Reactome database has sparse hierarchical relationships between pathway entities. Fitting complex multi-omics data using a neural network constructed from Reactome’s pathway hierarchy is challenging. For further mining of the hierarchical relationships of the latent pathway entities, the pathway entities were represented as nodes, and the genes of the entities were represented as features in this experiment.

The matrix $H\in{R}^{m\ast n}$ is constructed for the feature representation of the pathway nodes. *m* and *n* denote the number of nodes and the feature dimension of the node, respectively. $A\in{R}^{m\ast m}$ is the graph adjacency matrix constructed from known pathway hierarchy relationships. The GCN is defined as follows:


(5) \begin{equation*} {H}^{\left(l+1\right)}=\mathrm{\sigma} \left({\hat{D}}^{\frac{-1}{2}}\ \overset{\sim }{A}\ {\hat{D}}^{\frac{-1}{2}}\ {H}^{(l)}\ {W}^{(l)}\right) \end{equation*}

where ${H}^{(l)}$ denotes the feature input of the layer $l$. ${W}^{(l)}$ denotes the training parameter of the layer $l$. $\overset{\sim }{A}$ denotes the adjacency matrix and $\hat{D}$ denotes the degree matrix of $\overset{\sim }{A}$.

In the experiment, only latent predicted relationships were raised to the original topology to ensure the original biological pathway structure. Let ${\hat{h}}_i^j\ \mathrm{and}\ {\hat{h}}_{i-1}^k\in{R}^n$ be the feature representation of the *j*th node on layer *i* and the *k*th node on layer $i-1$, respectively, obtained by graph neural network aggregation. Also, *n* is the dimensionality of the input through the graph neural network. ${\hat{H}}_{\mathrm{connet}}\left({\hat{h}}_{i-1}^{\mathrm{k}},{\hat{h}}_i^{\mathrm{j}}\right)$ denotes if there is a new relationship between two pathway entities, which is defined as follows:


(6) \begin{equation*} {\hat{H}}_{\mathrm{connet}}\left({\hat{h}}_{i-1}^k,{\hat{h}}_i^j\right)=\left\{\ \begin{array}{c}1,\ \mathrm{if}\ \mathrm{sigmoid}\left({\hat{h}}_{i-1}^k\ast{\hat{h}}_i^j\right)>0.9\\{}\ 0,\ \mathrm{otherwise}\end{array}\right.. \end{equation*}

The new pathway entity feature representation is the inner product, and the inner product result is fed into the sigmoid function to calculate the final prediction probability. Only the pathway connections with a predicted probability of more than 0.9 were retained in the link prediction to make the network’s hierarchical relationship more representative.

### Interpretability of the model

DeepLIFT is an interpretable method to assign an importance score to the inputs for a given output using ‘reference activation’. DeepLIFT compares the activation of each neuron with its ‘reference activation’ and assigns an importance score based on the difference. In this study, the importance score was calculated using the ‘Rescale Relu’ of DeepLIFT. Supposing the *k* layers have a set of input neurons $\Big\{{x}_1,$…$, {x}_n\Big\}$, corresponding to an output *t*, the contribution ${C}_{x_i}^k$ of DeepLIFT to the node ${x}_i$ of *k*th layer is calculated as follows:


(7) \begin{equation*} {C}_{x_i}^k=\frac{\partial t}{\partial{x}_i}\ast \Delta t \end{equation*}

where ∆*t* = ($t$ − ${t}^0$) represents the difference-from-reference, and ${t}^0$ represents the reference activation of $t$. Then, the DeepLIFT is a locally interpretable method for only a particular sample.

The global interpretability method is provided by aggregating the contributions of a specific set of samples, which is defined as follows: 


(8) \begin{equation*} S{C}_{x_i}^k=\sum_{j=1}^s{C}_{x_{ij}}^k \end{equation*}

where *s* represents the sample size and $S{C}_{x_i}^k$ represents the total importance score of node ${x}_i$ in *k*th layer.

All zeros were chosen as the starting input reference, where the reference for all neurons in the network can be computed based on the input reference and network propagation.

### Cell culture

The human bronchial epithelial cell line 16HBE and the LUAD cell lines H1975, A549, CALU-3 and PC9 were sourced from MEISEN CELL (Meisen CTCC, China) and verified through STR analysis. Mycoplasma contamination was monitored regularly. The cells were grown in a high-sugar DMEM with 10% FBS, supplied by Viva Cell BIOSCIENCES (XP Biomed Ltd, China), at 37°C and 5% CO_2_ in a humid environment.

### siRNA and plasmid transfection

H1975 and CALU-3 LUAD cells were transfected using UltraFection 3.0 (4A BIOTECH, China) with NC, PSMC1 (siPSMC1) or PSMD11 (siPSMD11) siRNA (ykang, China). The siRNA sequences can be found in Table S1.

#### Reverse transcription and semi-quantitative PCR

Total RNA was extracted and purified with TRIzol (Cwbio, China) following the manufacturer’s protocol. mRNA reverse transcription was done using Hifair® II 1st Strand cDNA Synthesis Kit (Yeasn, China). Quantitative PCR was conducted using specific primers for PSMC1 and PSMD11 genes, designed with Primer 3 software. The expression of target genes was normalized with actin as an internal control. The primer pair sequences are in Table S2.

#### Western blot (WB) and antibodies

Proteins were extracted from cells according to Beyotime’s protocol. Protein concentrations were quantified using the BCA Protein Assay Kit (Solarbio, China). The WB was performed as previously reported [26].

#### Cell proliferation, transwell, and wound healing assays

The CCK-8 (Solarbio, China), colony formation, transwell and wound healing assays were performed according to previous studies [27–29].

#### IC50 measurement using CCK-8 assay

Cells were inoculated in 96-well plates with 5000 cells per well. After adherence, cells were treated with gradient concentrations of afatinib for 24 h. H1975 treatment concentrations included 0, 1, 2.5, 5, 10, 25, 50 and 100 nM. On the other hand, CALU-3 treatment concentrations included 0, 0.125, 0.25, 0.5, 1, 2.5, 5 and 10 nM. The medium was removed, washed once with PBS, and 100 μl of 10% CCK-8 solution was added to each well. After 3 h of incubation at 37°C, the OD at 450 nm was measured using a UV spectrophotometer. The software GraphPad Prism 8.0 was used for IC50 calculation.

## RESULT

### Classification performance of IBPGNET in LUAD postoperative recurrence

**Figure 1:**
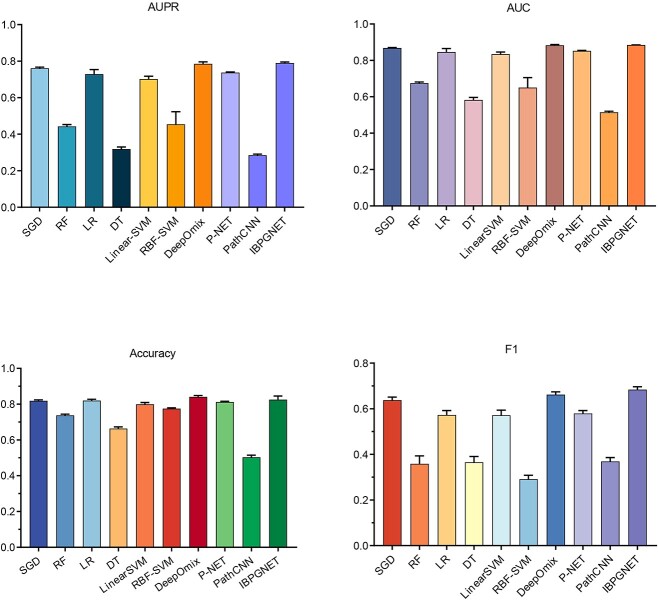
The performance comparison of SGD, RF, LR, DT, LinearSVM, RBF-SVM, DeepOmix, P-NET, PathCNN and IBPGNET.

To demonstrate the effectiveness of IBPGNET, we compared it with other classification algorithms, including random gradient descent (SGD), random forest (RF), logistic regression (LR), decision tree (DT), linear-SVM, RBF-SVM and P-NET [19], PathCNN [20] and DeepOmix [21]. The area under curve (AUC), area under the precision-recall curve (AUPR), accuracy and F1 score (F1) were used for evaluating the experimental results. Finally, to ensure the stability of the experiment, we performed 5-fold cross-validations and repeated the experiment 5× to reduce the effect of randomness. The final evaluation score was calculated by averaging the results of the five experiments. As shown in Figure 1, IBPGNET achieved an AUPR of 0.790 in 5-fold cross-validation, which is better than the other methods (SGD: 0.761, RF: 0.442, LR: 0.729, DT: 0.318, linear-SVM: 0.701, RBF-SVM: 0.454, DeepOmix: 0.785, P-NET: 0.737, PathCNN: 0.285). In addition, IBPGNET had an AUC of 0.88, an accuracy of 0.82 and an F1 of 0.68. These results show that IBPGNET outperforms other classification methods.

### Performance of IBPGNET on different omics data types

**Figure 2:**
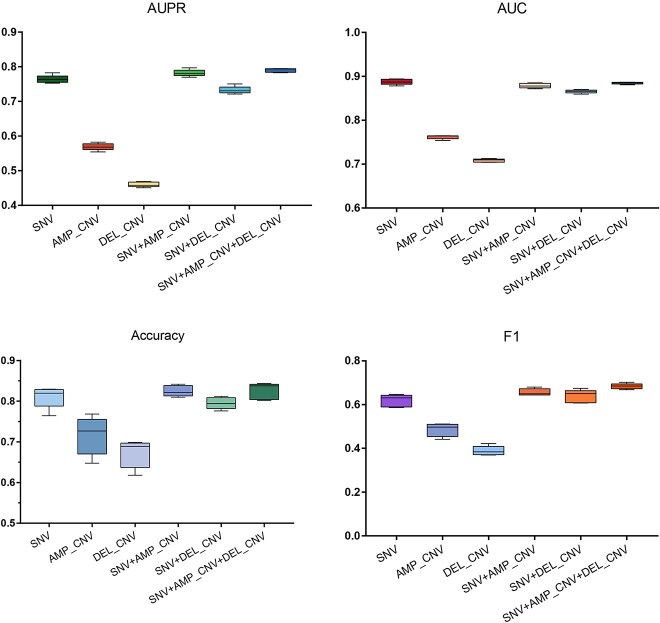
Performance of IBPGNET on different omics data types.

We evaluated IBPGNET flexibility on integrative multi-omics data for improving prediction performance. The CNV data and SNV data were used in this experiment. CNV data was divided into two subsets: AMP_CNV and DEL_CNV. In the experiments, we compared IBPGNET performance across different types of omics data, including SNV, AMP_CNV, DEL_CNV, SNV + AMP_CNV, SNV + DEL_CNV and SNV + AMP_CNV + DEL_CNV. As shown in Figure 2, integrating three omics data types outperformed the other omics data integrations. It achieved an AUPR of 0.79 in IBPGNET, which is better than the other omics data integrations (SNV: 0.76, AMP_CNV: 0.56, DEL_CNV: 0.45, SNV + AMP_CNV: 0.78, SNV + DEL_CNV: 0.73). In addition, the results showed that SNV performed better than AMP_CNV and DEL_CNV in predicting LUAD recurrence, indicating that the postoperative recurrence of LUAD is strongly associated with SNV.

### The effect of latent biological pathway relationships in IBPGNET

**Figure 3:**
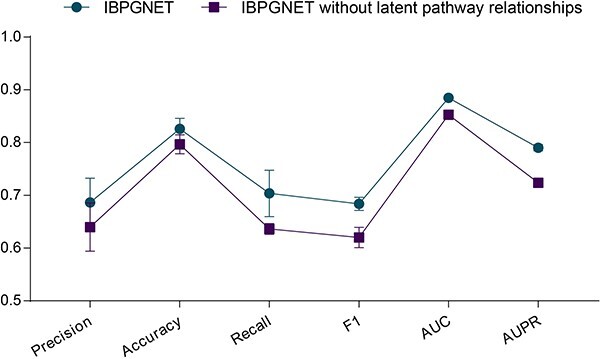
The effect of latent pathway hierarchy relationship on IBPGNET.

Pathway entities, including nucleic acids, proteins, complexes, vaccines, anti-cancer therapeutics and small molecules, form a network of biological interactions grouped into pathways [19]. In this experiment, IBPGNET is constructed based on existing pathway hierarchy relationships, which can provide excellent interpretability for the deep learning model. However, the sparse pathway hierarchy relationship makes it difficult to fit complex LUAD recurrence tasks. Therefore, a pathway graph neural network is proposed for latent pathway hierarchy relationship prediction, which can provide more information for LUAD recurrence prediction and help mining latent pathway hierarchy relationships. To demonstrate the role of the GCN-generated pathway hierarchy relationships in the prediction performance, we compared IBPGNET performance with and without latent hierarchy relationships. As shown in Figure 3, the IBPGNET with latent hierarchy relationships performed better in LUAD recurrence prediction than the IBPGNET without latent hierarchy relationships. Besides, these latent hierarchy relationships can provide insights for discovering new biological pathway relationships.

### The interpretability of IBPGNET

**Figure 4:**
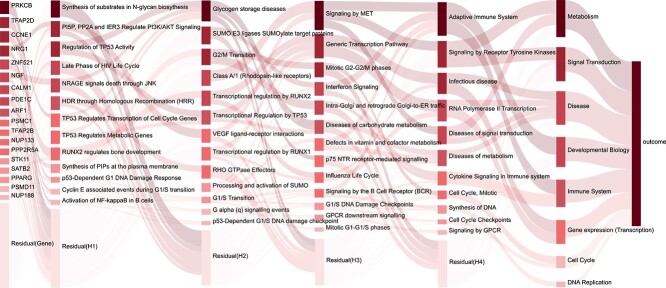
Visualization of biological hierarchies in IBPGNET.

To show the structure of IBPGNET, we visualized the scores of nodes in each layer of IBPGNET for LUAD recurrence prediction. The IBPGNET structure’s node size and color intensity systematically denote the omics features’ significance and predictive capacity for LUAD recurrence, with larger, darker nodes reflecting greater importance and influence on model predictions, offering a visual and hierarchical depiction of critical elements. The results are shown in Figure 4. The nodes on the left represent the input omics features, the following few layers represent higher-level biological entities and the last layer represents the model output. By visualizing the IBPGNET structure, we can understand the recurrence regulatory relationship in LUAD.

IBPGNET constitutes a range of molecules and pathways, including cell cycle, cell death, signal transduction, transcriptional regulation, metabolic and cell secretion pathways. Cell cycle-related proteins play essential roles in the recurrence and metastasis of LUAD [30]. For example, overexpression of Cyclin D1, CDK4, CDK6 and the deletion of p16 and p27 are closely associated with LUAD’s malignancy and prognosis [31, 32]. In addition, deletion or overexpression of cell cycle checkpoint proteins has been associated with LUAD’s risk of recurrence and metastasis [33]. Abnormalities in glucose metabolism involving the Warburg effect, glycosylation modifications and their associated inhibitors were shown to inhibit the growth and metastasis of LUAD cells [34].

We may learn more about the mechanisms of LUAD development and metastasis by evaluating the gene strata and determining the contribution of these genes to model prediction. Therefore, we looked at the gene strata and estimated an overall importance score for each gene to determine their relevance in the model’s predictions. The known LUAD driver genes PRKCB, CCNE1, NRG1, ZNF521 and NGF, previously linked to metastatic illness, were highly rated genes [35–37]. In addition, less common genes, such as CALM1, PSMC1, PSMD11 and PDE1C, significantly improved prediction accuracy. IBPGNET bridges the research gap by melding novel insights with established LUAD pathogenesis and metastasis literature, enhancing our model’s robustness and situating it within the broad scope of scientific research, thus highlighting our contribution to the nuanced understanding of LUAD, backed by authoritative sources.

### Functional validation of PSMC1 and PSMD11 in LUAD

**Figure 5:**
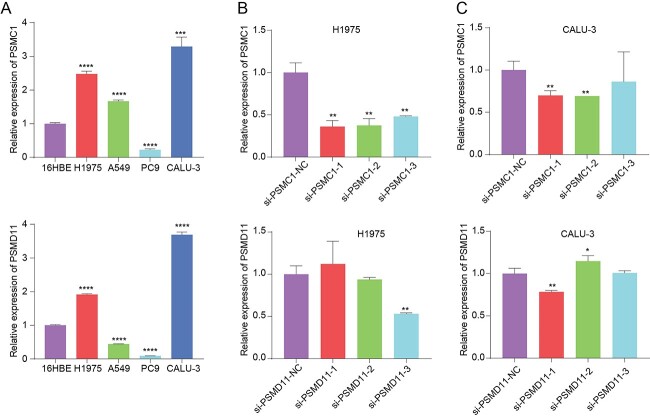
Expression of PSMC1 and PSMD11 and siRNA study. (**A**) Expression of PSMC1 and PSMD11 in the bronchial epithelial cell line 16HBE and the LUAD cell lines. (**B**) H1975 cells transfected with control siRNA or PSMC1/PSMD11 siRNA. (**C**) CALU-3 cells transfected with control siRNA or PSMC1/PSMD11 siRNA.

We compared the expression levels of PSMC1 and PSMD11 in the bronchial epithelial cell line 16HBE and the LUAD cell lines H1975, A549, PC9 and CALU-3. We observed that the expression levels of PSMC1 and PSMD11 in H1975 and CALU-3 were higher than in normal 16HBE cells (Figure 5A). This differential expression suggests a potential role for these proteins in LUAD pathogenesis. Then, we transfected one pair of negative control (NC) and three pairs of siRNA into the above cells to obtain si-PSMC1 and si-PSMD11 knockdown cells. The knockdown efficiency was evaluated, resulting in successful downregulation of PSMC1 and PSMD11 in si-PSMC1–2 and si-PSMD11–1 transfected cells, respectively (Figure 5B and C).

**Figure 6:**
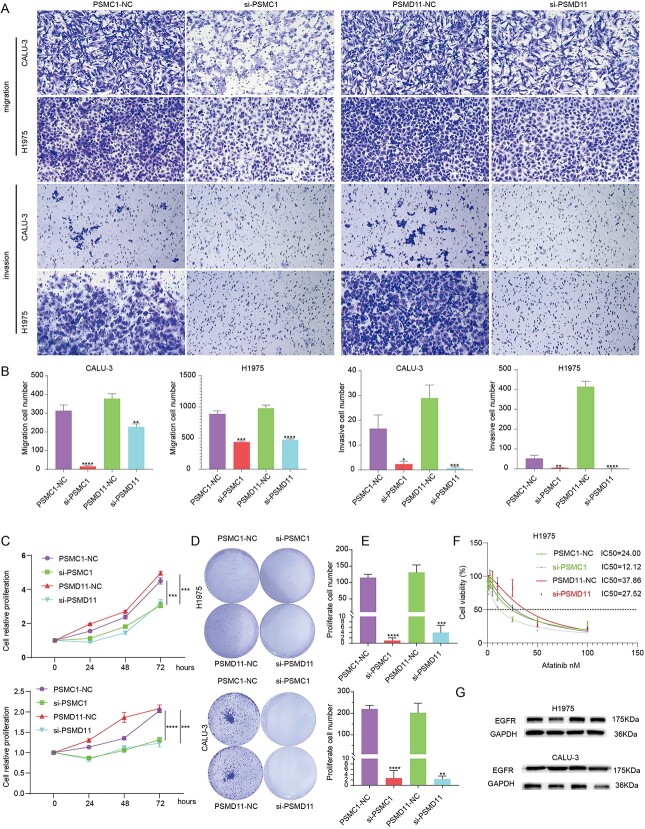
Functional validation of PSMC1 and PSMD11 in LUAD. (**A**) The migratory and invasive capabilities of the H1975 and CALU-3 cells were evaluated by using Transwell migration and invasion assays. (**B**) Quantification of migration and invasion cells showed that the cell migration and invasion capacities in si-PSMC1 and si-PSMD11 group were remarkably inhibited. (**C**) si-PSMC1 and si-PSMD11 reduced H1975 and CALU-3 cells proliferation by CCK-8 assay. (**D**) Clonogenic assays were carried out for H1975 and CALU-3 cells transfected with either si-PSMC1 or si-PSMD11. E.si-PSMC1 and si-PSMD11 reduced H1975 and CALU-3 cells proliferation by clonogenic assays. (**F**) The IC50 of si-PSMC1 and si-PSMD11 were analyzed by the CCK8 assay. (**G**) WB analysis of the expression of EGFR protein in H1975 and CALU-3 cells.

Cell phenotypic evaluation showed reduced migration, invasion and proliferation of H1975 and CALU-3 cells transfected with si-PSMC1 and si-PSMD11 (Figure 6A–E). These outcomes suggest that PSMC1 and PSMD11 play vital roles in the aggressive phenotypes of LUAD cells. Afatinib is a tyrosine kinase inhibitor (TKI) that targets the epidermal growth factor receptor (EGFR) and has been approved for treating LUAD. However, despite initial clinical benefits, many patients with LUAD resist afatinib and experience disease recurrence [38]. To explore the implications of PSMC1 and PSMD11 knockdown on drug sensitivity, we utilized the Cell Counting Kit-8 (CCK-8) assay to determine the half-maximal inhibitory concentration (IC50) of each cell group after treatment with different concentrations of afatinib. The results showed that the tolerance of H1975 cells to afatinib decreased after transfection with si-PSMC1 and si-PSMD11 (Figure 6F). This result underscores the potential of targeting these genes to overcome drug resistance in LUAD treatment. EGFR is a crucial protein involved in the development and recurrence of LUAD. EGFR overexpression or mutation increases tumor motility, invasiveness and resistance to chemo and radiation therapies [39]. Agents specifically targeting EGFR have proven effective in treating advanced non-small cell lung cancer and other diseases with EGFR mutations [40]. A WB was used to detect the expression of EGFR in each group of cells after transfection. The expression of EGFR in H1975 and CALU-3 cells transfected with si-PSMC1 and si-PSMD11 was significantly reduced compared to NC-transfected cells (Figure 6G). This reduction in EGFR expression could elucidate the mechanism behind the observed decrease in tumor aggressiveness and increased afatinib sensitivity, highlighting the therapeutic potential of inhibiting PSMC1 and PSMD11 in LUAD.

## DISCUSSION

Despite the tremendous developments in LUAD treatment, the prognosis of patients remains poor due to tumor recurrence and metastasis plagues that result from late diagnosis and the development of treatment resistance. Lung cancer treatment is currently guided by intrinsic tumor characteristics, including histology, altered drivers, PD-L1 and TMB. In addition, extrinsic tumor characteristics, such as immune infiltration, are gradually being integrated into treatment regimens to improve the treatment of lung cancer patients [41]. Among the alternatives, personalized medicine that combines neural network modeling approaches with *in vitro* molecular experiments is very encouraging. Combining these approaches with biomarker identification can significantly improve NSCLC treatment and patient survival.

The continuous development of high-throughput sequencing technologies allowed researchers to obtain large-scale omics data from different molecular levels, including genomics, transcriptomics, proteomics and metabolomics [42]. These data can promote a deep understanding of biological processes and molecular mechanisms in the microcosm. Previously, there were certain obstacles in the integrative analysis of omics data due to their heterogeneity, high dimension and small sample sizes. In addition, it is challenging to understand deep learning at the semantic level, and the lack of mathematical tools to evaluate the network’s feature representation ability limits its development in the biological field. However, the above problems have been solved with the availability of pathway hierarchy relationships and detailed gene annotations at an unprecedented scale and speed. To this end, we propose IBPGNET, a neural network based on pathway hierarchy relationships, for LUAD recurrence prediction. IBPGNET is a restricted neural network model that can only transmit information according to specific biological connections so that it can simulate the flow of information at the molecular level. IBPGNET also effectively identified meaningful potential biomarkers associated with LUAD recurrence through information backpropagation.

Compared to fully connected networks with the same nodes, IBPGNET has fewer parameters, which significantly reduces the training time and effectively prevents overfitting. Besides, a pathway graph neural network can predict latent pathway associations based on the original pathway hierarchy relationship topology. It has been demonstrated that the latent pathway relationships generated by GCN prevent original topology destruction and achieve excellent performance in LUAD recurrence prediction compared to the original pathway structure. To further understand the internal regulation of LUAD recurrence, we visualized the whole network structure of IBPGNET used in LUAD recurrence.

IBPGNET, a multi-omics data analysis network, can accurately predict advanced LUAD’s developmental trend and potential biochemical relapse based on patients’ data. The structure visualization of IBPGNET enables a more comprehensive understanding of the involved biological pathways and processes, providing in-depth insights to conduct future research. Researchers can explore potential biological processes involved in LUAD progression through the predictive results of IBPGNET, which can be translated into therapeutic opportunities to help patients achieve better treatment outcomes.

IBPGNET re-identified known LUAD-associated genes, such as PRKCB, CCNE1, NRG1, ZNF521 and NGF, characterizing a series of related molecular pathways. In addition, IBPGNET identified PSMC1 and PSMD11 as the two relevant genes in LUAD, which we validated experimentally. These genes may provide a therapeutic basis for patients with metastatic LUAD in the PSMC1 and PSMD11 transcriptional component layers. Specifically, the expression levels of PSMC1 and PSMD11 were significantly higher in LUAD cells, H1975 and CALU-3 than in normal cells. After the individual knockdown of PSMC1 and PSMD11, the sensitivity of LUAD cells to afatinib was elevated, and cell migration, invasion and proliferation were decreased. At the same time, EGFR expression was significantly reduced, suggesting that PSMC1 and PSMD11 may regulate cellular sensitivity to afatinib through EGFR expression.

In conclusion, IBPGNET, a neural network based on the hierarchy of biological pathways, successfully predicted the recurrence of LUAD and identified potential biomarkers of LUAD recurrence through information backpropagation. The structural visualization of IBPGNET provides deeper insights into the biological pathways associated with LUAD progression, which can translate into therapeutic opportunities for achieving better diagnostic and treatment outcomes.

**Key Points**

- IBPGNET: A neural network leveraging pathway hierarchy for LUAD recurrence prediction.
- IBPGNET simulates molecular information flow through specific biological connections.
- Multi-omics data tool IBPGNET predicts advanced LUAD trends and biochemical relapse.
- IBPGNET’s visualization deepens understanding of biological pathways in LUAD.
- Predictions by IBPGNET spotlight therapeutic opportunities for improved LUAD treatment.

## Supplementary Material

Table_S1_bbae080

Table_S2_bbae080

fig_S1_bbae080

## Data Availability

Data can be accessed in a public repository, and all pertinent study data are either in the article or provided as supplementary material. IBPGNET is available on GitHub at https://github.com/lanbiolab/IBPGNET.

## Author Biographies

**Zhanyu Xu** received his PhD in Surgery from Guangxi Medical University and is now a cardiothoracic surgeon at the First Affiliated Hospital of Guangxi Medical University. His main research interests are molecular markers and mechanisms in the recurrence and metastasis of lung adenocarcinoma.

**Haibo Liao** received the B.S. degree in software engineering from Guangxi University of Science and Technology, China, in 2021. He is currently a MSc student in computer technology, Guangxi University from 2021. His research interests include bioinformatics and deep learning.

**Liuliu Huang** is a PhD candidate in surgery at Guangxi Medical University and an associate chief physician of cardiothoracic surgery at the First Affiliated Hospital of Guangxi Medical University. His research focuses on minimally invasive treatment of congenital heart disease.

**Qingfeng Chen** received the Ph.D. degree in computer science from the University of Technology Sydney in 2004. He is currently a professor and the director of the bioinformatics team of the State Key Laboratory for Conservation and Utilization of Subtropical Agro-Bioresources at Guangxi University, China. His research interests include bioinformatics, data mining, and artificial intelligence.

**Wei Lan** received the Ph.D. in computer science from Central South University, China, in 2016. Currently, he is lecturer in School of Computer, Electronic and Information in the Guangxi University, Nanning, China. His current research interests include bioinformatics and machine learning.

**Shikang Li** received his PhD in Surgery from Wuhan University and is currently a Professor of Cardiothoracic Surgery at the First Affiliated Hospital of Guangxi Medical University, with a focus on precision diagnosis and treatment of lung cancer as well as immunotherapeutic mechanisms.
