# Supplementary material for: IBPGNET: lung adenocarcinoma recurrence prediction based on neural network interpretability
Source: Brief Bioinform. 2024 Mar 31;25(3):bbae080. doi: 10.1093/bib/bbae080 (PMC10982951; doi:10.1093/bib/bbae080)
Supplement: Table_S1_bbae080 [file table_s1_bbae080.doc]

Table S1 siRNA sequences used in this work.

| SiRNA ID | Sequences（5’→3’） |
| --- | --- |
| si-PSMC1-1 | Forward AGGAUGACAAGGACAAGAAAAAG |
|  | Reverse CUUUUUCUUGUCCUUGUCAUCCU |
| si-PSMC1-2 | Forward AUCGUGUUUAUUGAUGAAAUUGA |
|  | Reverse UCAAUUUCAUCAAUAAACACGAU |
| si-PSMC1-3 | Forward CCGCAUUGACAGGAAGAUUGAGU |
|  | Reverse ACUCAAUCUUCCUGUCAAUGCGG |
| PSMC1-NC | Forward AGGUUGAAAAGCACACGAACAAC |
|  | Reverse GUUGUUCGUGUGCUUUUCAACCU |
| si-PSMD11-1 | Forward GCGUGACAUUCAGGAAAACGAUG |
|  | Reverse CAUCGUUUUCCUGAAUGUCACGC |
| si-PSMD11-2 | Forward GUCAGAGAAAAGAACUUUCUUAC |
|  | Reverse GUAAGAAAGUUCUUUUCUCUGAC |
| si-PSMD11-3 | Forward UGGUGUCUUUGUACUUUGAUACC |
|  | Reverse GGUAUCAAAGUACAAAGACACCA |
| PSMD11-NC | Forward GCGCGACGUUCCGGAGAACCAUA |
|  | Reverse UAUGGUUCUCCGGAACGUCGCGC |
