# Supplementary material for: IBPGNET: lung adenocarcinoma recurrence prediction based on neural network interpretability
Source: Brief Bioinform. 2024 Mar 31;25(3):bbae080. doi: 10.1093/bib/bbae080 (PMC10982951; doi:10.1093/bib/bbae080)
Supplement: Table_S2_bbae080 [file table_s2_bbae080.doc]

Table S2 Primer sequences used in this study.

| Primes | Sequences（5’→3’） |
| --- | --- |
| GAPDH | Forward ACATCGCTCAGACACCATG |
|  | Reverse TGTAGTTGAGGTCAATGAAGGG |
| PSMC1 | Forward ATGTGATTCCTGGCTCCATCTC |
|  | Reverse CGGCACTGAGTGTGAGGTGT |
| PSMD11 | Forward TAGGTGAGTGCTGGCTTCAG |
|  | Reverse ACACACTCTCCAAAGGACCG |
