# Supplementary figures and images for: IBPGNET: lung adenocarcinoma recurrence prediction based on neural network interpretability
Source: Brief Bioinform. 2024 Mar 31;25(3):bbae080. doi: 10.1093/bib/bbae080 (PMC10982951; doi:10.1093/bib/bbae080)

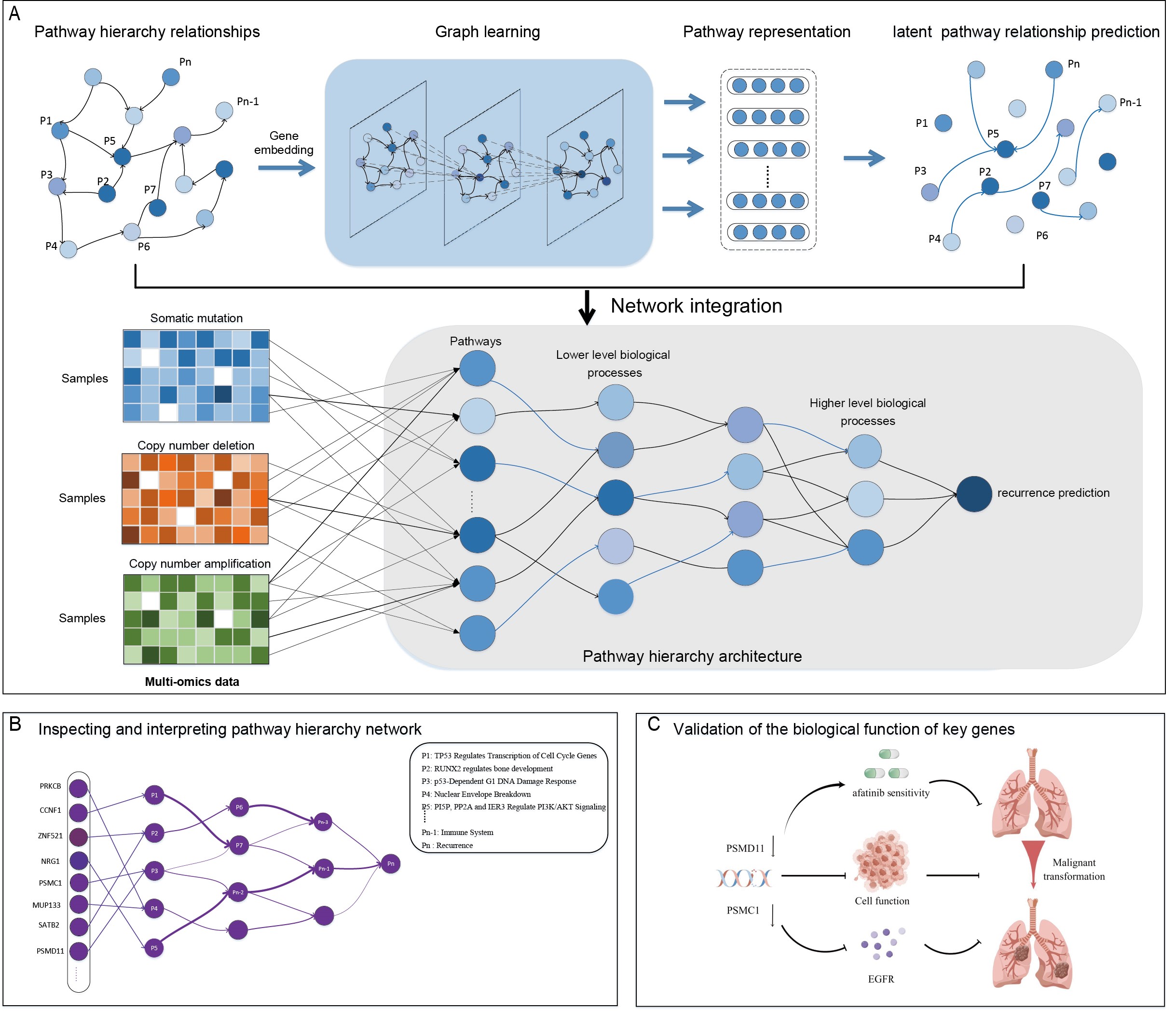

Supplement: fig_S1_bbae080 [file fig_s1_bbae080.jpeg]
